# Direct Unequal Cleavages: Embryo Developmental Competence, Genetic Constitution and Clinical Outcome

**DOI:** 10.1371/journal.pone.0166398

**Published:** 2016-12-01

**Authors:** Qiansheng Zhan, Zhen Ye, Robert Clarke, Zev Rosenwaks, Nikica Zaninovic

**Affiliations:** Ronald O. Perelman and Claudia Cohen Center for Reproductive Medicine, Weill Cornell Medicine, New York, New York, United States of America; University Hospital of Münster, GERMANY

## Abstract

**Objective:**

To investigate the prevalence, developmental potential, chromosomal constitution and clinical outcome of embryos with direct unequal cleavages (DUC).

**Design:**

A retrospective observational study.

**Setting:**

Academic Institution.

**Participant:**

21,261 embryos from 3,155 cycles cultured in EmbryoScope^®^.

**Results:**

The total incidence of DUCs per embryo occupying the first three cleavages were 26.1%. Depending of the cell stage, DUC rate was 9.8% at first cleavage (DUC-1), 9.1% at second cleavage (DUC-2), and 3.7% at third cleavage (DUC-3) with 3.6% of embryos exhibiting multiple DUCs (DUC-Plus). The occurrence of DUCs was not correlated with female gamete age or source. The incidence of DUC-1 was significantly higher in embryos fertilized by epididymal and testicular sperm (13.6% and 11.4%, respectively) compared to ejaculated sperm (9.1%, all p<0.05). The total incidences of DUCs were strongly correlated with the onset of blastomere multinucleation (MNB) during the first three divisions. In MNB embryos, DUCs incidence are two to three times more likely to develop when compared to non-MNB embryos (OR = 3.11, 95% CI [2.64, 3.67] at 1-cell stage, OR = 2.64, 95% CI [2.39, 2.91] at 2-cell stage and OR = 2.51, 95% CI [1.84, 3.43] at 4-cell stage). The blastocyst formation rates gradually decreased from 61.0% in non-DUC to 40.2% in DUC-3, 18.8% in DUC-2, 8.2% in DUC-1 and 5.6% in multiple DUC embryos (DUC-Plus). The known implantation rates (FH) for day 3 (D3) transfers were 12.42% (n = 3172) in Non-DUC embryos, 6.3% (n = 127) in DUC-3, and 2.7% (n = 260) in DUC-2 embryos. No live births resulted from either DUC-1 (n = 225) or DUC-Plus (n = 100) embryo transfers. For blastocyst transfers, lower implantation rates (33.3%) but similar live birth (LB) rates (40%) were observed if DUC blastocysts were transferred. Comparatively rates in Non-DUC blastocyst were 45.2% and 34.8%, respectively. The euploid rate gradually increased from DUC-1, -2, -3 to Non-DUC (13.3%, 19.5%, 33.3%, 45.6%, p<0.001) for D3 biopsied embryos. Interestingly, the trend of decreased euploidy disappeared in DUC D5/6 biopsied embryos and similar rates were exemplified in DUC (D5 56.3%, D6 35.6%) vs. non-DUC (D5 51.4%, D6 33.8%) embryos.

**Conclusion:**

Blastocyst formation, implantation potential and euploid rate were significantly reduced in DUC embryos. DUC embryos should be deselected for D3 transfers, but should be culture to blastocyst stage for possible ET.

## Introduction

Normal mitotic cell division results in two daughter cells. However, abnormal mitosis resulting in three or more daughter cells has been observed in common cancers [1, 2], cells infected with papovirus[3] and the placenta[4]. This phenomenon has also been referred to as trichotomic mitosis [5], tripolar or multipolar mitosis [6], direct cleavage [7] or abnormal cleavage. The duration of the blastomere cell cycle is usually around 10 to 12 hours [8], which should be sufficient for the embryo to undergo two consecutive cytokinesis and cell genome replication. Extremely short cell cycles with an incomplete DNA replication may be associated with unequal distribution of DNA to blastomeres [9, 10]. We have termed this phenomena direct unequal cleavage (DUC). By using a time-lapse monitoring system, DUC was define as the abrupt cleavage of one blastomere into three daughter blastomeres or an interval of cell cycles less than five hours. It has also been reported that unequal cleavages are common in tripronuclear human oocytes [11–13]. In 2PN human embryos, DUC-1 (at the 1^st^ cleavage) was reported with large variations in frequency ranging from 8.3% to 26% [10, 14–16]. DUCs were observed at the 2^nd^ cleavage stage with similar frequency 17–18% [5, 14]. This abnormal mitosis suggests that the occurrence of tripolar mitosis can impair early embryo development in human two-pronuclear embryos [5, 10, 14, 17]. A correlation between the occurrence of DUCs, impaired embryo development, and implantation potential was observed in both animal and human embryos [5, 9, 10, 12–14, 17]. However, sample size in these studies were small and did not include information about clinical outcome or chromosomal status.

Time-lapse cinematography provides an uninterrupted evaluation of embryo morphological and dynamic parameters. The innovation of a practical time-lapse culturing system not only provides a great research tool for studying early embryo development, it can also be used to improve clinical outcome [8, 9]. We assembled a time-lapse prototype system in 2005[18] to study mouse and human embryo development but the application was limited to clinical research. Single embryo transfers are an efficient strategy to reduce multiple pregnancies, but accurate assessment of embryo developmental potential remains an essential challenge. Conventional embryo selection is based on static morphological grading systems, which may limit accurate embryo assessment. In order to detect and study DUCs in human embryos, continuous monitoring is required.

In the present study, we performed a retrospective systemic analysis focusing on the prevalence of DUCs in human IVF embryo. Our study concentrated on the association of DUCs with embryo developmental potential, clinical outcome and chromosomal constitution with the aim of applying these findings to future embryo selection.

## Materials and Methods

### Study design and participants

This is a retrospective cohort study conducted in an academic institution from November 2011 to June 2014. Only two-pronuclear (2PN) embryos cultured in time-lapse incubators (EmbryoScope^®^, Vitrolife, Sweden) were included in the study. A total of 21,261 embryos from 3,155 cycles (2471 ICSI and 684 standard IVF) were analyzed.

### Ethical approval

This retrospective cohort analysis was conducted in accordance with a research protocol approved by the Committee of Human Rights Research Weill Cornell Medicine (IRB # 1304013779)**.**

### Fertilization, embryo culture

Following oocyte retrieval, oocytes were fertilized using standard IVF or ICSI according to patient indication. For ICSI, oocytes were loaded immediately after injection on day zero (D0) or the next day (D1). For standard IVF, 2PN embryos were loaded on D1. Oocytes or zygotes were individually loaded into pre-equilibrated culture slides (EmbryoSlide^®^, Vitrolife, Sweden), filled with 25μL of in-house sequential culture medium (C1 medium for D0 to D3) and covered with tissue culture oil. Embryos were cultured in the EmbryoScope^®^ (Vitrolife, Denmark) at 37°C, 5.8% CO_2_ and 5% O_2_. C2 media (D3 to D5) was changed on day 3 for blastocyst (BL) culture.

### Genetic diagnosis/screening

Preimplantation Genetic Diagnosis and Screening (PGD/PGS) biopsies were performed on D3, D5 or D6 embryos. For D3 biopsy, 1–2 blastomeres were obtained following Acid Tyrode’s or laser (ZILOS-tk^®^, Hamilton Thorne, USA) opening of the zona pellucida. For blastocyst (BL) biopsy, up to 10 trophectoderm (TE) cells were collected after laser hatching. Biopsied samples were analyzed via FISH, PCR, aCGH (BlueGnome 24sure, Illumina^®^, USA) or Single Nucleotide Polymorphism (Spectrum^®^, Natera, USA).

### Time-lapse microscope (TLM) image capture and annotation

Images were recorded automatically every 10 minutes with seven focal planes illuminated by red LED light (635 nm). The following time points were annotated: appearance of pronuclear (PN), syngamy (PN fading), time of division (2, 3, 4, 5, 6, 7, 8 and 9 or more cells), morula, cavitation, early, fully expanded and hatching blastocyst. The number of multinucleated blastomeres (MNB) in the first 3 cleavages, evenness of blastomere and fragmentation percentages were also recorded.

### DUC annotation and classification

DUCs were annotated if a single blastomere directly cleaved into three or more daughter blastomeres, or the interval between mother and daughter cell division was equal or less than 5 hours [9, 10]. Regardless of the size, only cells containing visible nuclei were considered as blastomeres, otherwise, they were annotated as fragment. All DUC annotations were confirmed by one most experienced embryologist. DUC embryos were further classified as: DUC-1, DUC-2 or DUC-3 dependent upon the cleavage stage when the DUCs occurred. DUC-1: abnormal cleavage occurred after syngamy (1-cell) resulting in 3–4 blastomeres. DUC-2: abnormal cleavage occurred at the 2-cell stage resulted in 5 or 6 blastomeres. DUC-3: abnormal cleavage occurred at the 4-cell stage resulting in 9 or more blastomeres (S1–S3 Videos). If DUCs occurred more than once, embryos were classified as DUC-Plus based on the earliest onset stage. DUC embryos were preferably excluded from the transfer on day 3 and day 5 except in cases where no other normally developing embryos were available.

### Clinical outcome measures

Embryo developmental outcome was measured at day 3 (D3) by cell stage and morphology, and at day 5 (D5) by blastocyst formation rates. Each embryo grade was determined by cleavage-stage and blastocyst-stage morphologic grading [19]. “Good” embryos on day 3 were classified as 8 or more cells and less than 20% fragmentation. Day 5, blastocysts were graded based on their expansion, inner cell mass and trophectoderm morphology[19]. Good blastocysts (2BB or higher) were considered for transfer or cryopreservation. Clinical pregnancy was confirmed within 6–8 weeks after transfer by the presence of the fetal heart. Known implantation data (KID) included only embryos from transfers in which all transferred embryos implanted or none implanted.

### Statistical analysis

Statistical analyses were performed using JMP^®^ Pro 11 software (SAS Institute Inc. USA). Chi-squared test or Logistic regression analyses were performed with *p*<0.05 considered to be statistically significant.

## Results

### Incidence and distribution of DUCs

**Fig 1:**
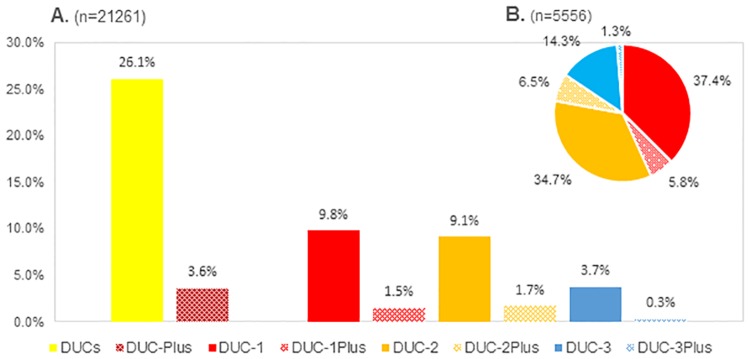
DUCs incidence and ratio. (A) DUCs incidence per embryo. (B) DUCs ratio in DUC embryos. DUC-1: direct unequal cleavage at 1st cleavage; DUC-1Plus: DUC occur more than once in DUC-1 embryos; DUC-2: direct unequal cleavage at 2nd cleavage; DUC-2Plus: DUC occur more than once in DUC-2 embryos; DUC-3: direct unequal cleavage at 3rd cleavage; DUC-3Plus: DUC occur more than once in DUC-3 embryos; Non-DUC: embryos without DUC; DUC-Plus: DUC occurred more than once (DUC-1Plus, DUC-2Plus and DUC-3Plus combined); DUCs: All DUC embryos.

DUCs occurred in 26.1% of all embryos with 3.6% of embryos exhibiting multiple episodes (DUC-Plus). The incidence of DUC-1 and DUC-2 were similar (9.8% and 9.1%) and were significantly higher than DUC-3 (3.7%, p<0.01) (Fig 1A). Out of all DUCs, DUC-1 and DUC-2 occurred more frequently then DUC-3 (34.7% and 37.4% vs. 14.3%, respectively) (Fig 1B). High DUC prevalence in IVF (n = 684) and ICSI cycles (n = 2471) was also confirmed by analyzing cycles with 4 or more 2PN embryos (n = 2383, 75.5% of all cycles). Of interest, in 71.1% cycles (ICSI 69.1%, IVF 78.5%) more than half of the embryos exhibited DUCs and only 0.1% (ICSI 0.1%, IVF 0.4%) were DUCs-free cycles. (S1 Fig).

### DUCs: gamete age and source

To study the correlation between DUCs and maternal age, embryos were stratified into 5 age groups according to the Society for Assisted Reproductive Technology (SART) guidelines[20]. The incidence of DUCs were similar in all oocyte (maternal) age groups (*p* = 0.18), as well as in paternal age groups (*p* = 0.19, Table 1). Furthermore, no differences were found between autologous and donor oocytes for DUCs incidence. (Table 1).

**Table 1:** DUCs incidence: gamete age, gamete source and oxygen concentration.

		n	DUC-1%(n)	DUC-2%(n)	DUC-3%(n)	DUC-Plus %(n)	Non-DUC %(n)
**Total**		**21261**	**9.8% (2080)**	**9.1% (1927)**	**3.7% (794)**	**3.6% (755)**	**73.9% (15705)**
**Oocyte Age**	<35	8987	10.0% (897)	9.2% (823)	3.5% (317)	3.3% (295)	74.1% (6655)
	35–37	4107	10.0% (409)	9.8% (401)	4.3% (177)	3.8% (154)	72.2% (2966)
	38–40	3749	9.7% (364)	8.8% (331)	4.0% (148)	3.8% (141)	73.8% (2765)
	41–42	1180	9.8% (116)	9.1% (107)	2.9% (34)	3.8% (45)	74.4% (878)
	>42	3238	9.1% (294)	8.2% (265)	3.6% (118)	3.7% (120)	75.4% (2441)
		*p-value*	*0*.*18*				
**Paternal Age**	<35	5717	10.0% (572)	9.2% (523)	3.8% (218)	3.6% (204)	73.5% (4200)
	35–37	3893	9.5% (371)	9.3% (361)	3.5% (135)	2.9% (112)	74.9% (2914)
	38–40	3801	10.5% (399)	9.3% (355)	3.6% (137)	3.5% (133)	73.1% (2777)
	41–42	1128	8.2% (93)	8.9% (100)	4.9% (55)	4.1% (46)	73.9% (834)
	>42	6722	9.6% (645)	8.8% (588)	3.7% (249)	3.9% (260)	74.1% (4980)
		*p-value*	*0*.*19*				
**Oocyte Source**	Autologous	19105	9.8% (1878)	9.1% (1729)	3.8% (718)	3.5% (674)	73.8% (14106)
	Donor	2156	9.4% (202)	9.2% (198)	3.5% (76)	3.8% (81)	74.2% (1599)
		*p-value*	*0*.*90*				
**Sperm Source**	**ICSI**	16046	9.3% (1487)	8.9% (1432)	3.8% (602)	3.5% (567)	74.5% (11958)
	Donor, frozen	1599	9.3% (149)	10.4% (166)	2.9% (46)	3.7% (59)	73.7% (1179)
	Ejaculate, frozen	1041	8.0% (83)	8.5% (88)	3.8% (40)	3.6% (37)	76.2% (793)
	Ejaculate, fresh	12285	**9.1%** (1118)	8.7% (1063)	3.9% (484)	3.5% (433)	74.8% (9187)
	Ejaculate combined (donor & husband, fresh & frozen)	14925	9.1% (1350)	8.8% (1317)	3.8% (570)	3.5% (529)	74.8% (11159)
	Epididymal sperm	419	**13.6%** (57)	11.7% (49)	2.4% (10)	3.1% (13)	69.2% (290)
	Testicular sperm	702	**11.4%** (80)	9.4% (66)	3.1% (22)	3.6% (25)	72.5% (509)
	**IVF**	5215	11.4% (593)	9.5% (495)	3.7% (192)	3.6% (188)	71.9% (3747)
	Ejaculate, frozen(husb,donor)	55	3.6% (2)	14.6% (8)	3.6% (2)	5.5% (3)	72.7% (40)
	Ejaculate	5160	**11.5%** (591)	9.4% (487)	3.7% (190)	3.6% (185)	71.8% (3707)
	*Frozen ejaculate*: *donor vs*. *husbandonor vs*.*husband*	*Pearson's chi-sq*.	*1*.*42*	*2*.*70*	*1*.*87*	*0*.*03*	*1*.*99*
		*p-value*	*0*.*23*	*0*.*10*	*0*.*17*	*0*.*86*	*0*.*16*
		*Odds Ratio*	*1*.*19*	*1*.*25*	*0*.*74*	*1*.*04*	*0*.*88*
	*Ejaculate*: *Frozen vs*. *Fresh*	*Pearson's chi-sq*.	*1*.*49*	*0*.*05*	*0*.*02*	*0*.*00*	*0*.*99*
		*p-value*	*0*.*22*	*0*.*83*	*0*.*88*	*0*.*96*	*0*.*32*
		*Odds Ratio*	*0*.*87*	*0*.*97*	*0*.*97*	*1*.*01*	*1*.*08*
	*Epididymal vs*. *Testicular sperm*	*Pearson's chi-sq*.	*1*.*19*	*1*.*50*	*0*.*53*	*0*.*17*	*1*.*39*
		*p-value*	*0*.*27*	*0*.*22*	*0*.*47*	*0*.*68*	*0*.*24*
		*Odds Ratio*	*1*.*22*	*1*.*28*	*0*.*76*	*0*.*87*	*0*.*85*
	*Ejaculate combined vs*. *Epididymal sperm*	*Pearson's chi-sq*.	*10*.*17*	*4*.*14*	*2*.*30*	*0*.*23*	*6*.*64*
		*p-value*	***<0*.*001***	*0*.*04*	*0*.*13*	*0*.*63*	*<0*.*001*
		*Odds Ratio*	*0*.*63*	*0*.*73*	*1*.*62*	*1*.*15*	*1*.*32*
	*Ejaculate combined vs*. *Testicular sperm*	*Pearson's chi-sq*.	*4*.*46*	*0*.*28*	*0*.*86*	*0*.*00*	*1*.*81*
		*p-value*	*0*.*03*	*0*.*60*	*0*.*35*	*0*.*98*	*0*.*18*
		*Odds Ratio*	*0*.*77*	*0*.*93*	*1*.*23*	*1*.*00*	*1*.*12*
	*Ejaculate sperm*: *ICSI (combined) vs*. *IVF*	*Pearson's chi-sq*.	*25*.*47*	*1*.*77*	*0*.*20*	*0*.*02*	*17*.*07*
		*p-value*	***<0*.*001***	*0*.*18*	*0*.*66*	*0*.*89*	*< 0*.*001*
		*Odds Ratio*	*0*.*77*	*0*.*93*	*1*.*04*	*0*.*99*	*1*.*16*
**O2%**	20%(Ambient)	434	11.3% (49)	9.7% (42)	1.2% (5)	1.8% (8)	76.0% (330)
	5%	14485	9.0% (1301)	8.8% (1275)	3.9% (564)	3.6% (521)	74.7% (10824)
		*Pearson's chi-sq*.	*2*.*74*	*0*.*40*	*8*.*66*	*3*.*78*	*0*.*38*
		*p-value*	*0*.*10*	*0*.*52*	*0*.*00*	*0*.*05*	*0*.*54*
		*Odds Ratio*	*1*.*29*	*1*.*11*	*0*.*29*	*0*.*50*	*1*.*07*

DUCs incidence: gamete age, gamete source and oxygen concentration. DUC-1: direct unequal cleavage at 1st cleavage; DUC-2: direct unequal cleavage at 2nd cleavage; DUC-3: direct unequal cleavage at 3rd cleavage; DUC-Plus: DUC occurred more than once. Non-DUC: embryos without DUC.

Correlation between DUC occurrence and sperm source were established by analyzing embryos generated by testicular, epididymal and ejaculate sperm using ICSI. Table 1 showed similar DUCs incidence in frozen sperm (husband vs. donor), husband ejaculate (fresh vs. frozen) and surgically retrieved sperm (testicular vs. epididymal). Incidences of DUC-2, DUC-3, or DUC-Plus were similar in ejaculate, epididymal and testicular sperm. However, DUC-1 incidence was significantly higher in epididymal (13.6%, *p* = 0.001) and testicular sperm (11.4%, *p* = 0.034) when compared to ejaculate sperm (9.1%).

### DUC and fertilization method

Only embryos fertilized by ejaculate sperm were included in this sub-study. No significant difference was observed between ICSI and IVF for DUC-2, DUC-3, or DUC-Plus embryos. However, DUC-1 occurred more often in IVF vs. ICSI embryos (11.5% vs. 9.1%, *p*<0.001, IVF Ejaculate vs. ICSI Ejaculate combined in Table 1).

To evaluate the correlation of oxygen concentration and DUC incidence, ICSI embryos using ejaculate sperm were analyzed: 434 in atmospheric 20% O_2_ vs. 14485 in 5% O_2_. The occurrence of DUC-1 and DUC-2 were slightly higher in atmospheric O_2_ (11.3% vs. 9.0% and 9.7% vs. 8.8%, *p* = 0.09 and *p* = 0.52) but the differences did not reach statistical significance (Table 1).

### DUC and multinucleation

In embryos exhibiting multinuclear blastomeres (MNB), we observed a higher occurrence of DUCs in subsequent cleavages (Table 2). DUCs incidence in MNB embryos are 2.5–3.1 folds higher compared to Non-MNB embryos in early cleavage stages (1-cell stage OR = 3.11, 95% CI [2.64, 3.67], 2-cell stage OR = 2.64, 95% CI [2.39, 2.91], and 4-cell stage OR = 2.51, 95% CI [1.84, 3.43], all p<0.001) (Table 2). The incidence of MNB after abnormal divisions (DUCs) were similar in DUC-2 and DUC-3 embryos when compared to Non-DUC embryos, but significantly lower in DUC-1 embryos compared to Non-DUC embryos (S1 Table).

**Table 2:** Incidence of DUCs per embryo and multinucleation.

	1-cell Stage	2-Cell Stage	4-Cell Stage
**Non-multinucleated embryos**	10.5% (1358/12954)	6.3% (809/12954)	3.3% (431/12954)
**Multinucleated embryos**	26.7% (219/820)	15.0% (1018/6804)	8.0% (47/591)
*Pearson Chi-Sq*.	*200*.*22*	*403*.*89*	*35*.*52*
*p-value*	*<0*.*001*	*<0*.*001*	*<0*.*001*
*Odds Ratio*	*3*.*11*	*2*.*64*	*2*.*51*
*95% CI*	*2*.*64–3*.*67*	*2*.*39–2*.*91*	*1*.*84–3*.*43*

DUCs: direct unequal cleavage.

To further investigate the correlation of multinucleated blastomere and DUCs, 7155 embryos cultured only in 5% O_2_ were analyzed (Table 3 study A). Similarly, the chance of DUCs were 2–9 folds higher in multinucleated blastomeres compared to mononucleated blastomeres even after the correction for the sperm source used (Table 3 study B).

**Table 3:** Incidence of DUCs per blastomere and multinucleated blastomere (MNB).

		1-Cell Stage	2-Cell Stage	4-Cell Stage
**Study A**	**MNB**	23.9% (85/356)	11.9% (447/3763)	10.9% (96/878)
	**Non-MNB**	7.8% (527/6799)	4.9% (537/10932)	1.3% (372/27999)
	**MNB %**	5	25.6	3
	*chi-sq*.	*112*.*46*	*217*.*46*	*492*.*62*
	*p-value*	*<0*.*001*	*<0*.*001*	*<0*.*001*
	*Odds Ratio*	*3*.*73*	*2*.*61*	*9*.*12*
	*95% CI*	*2*.*89–4*.*84*	*2*.*29–3*.*00*	*7*.*21–11*.*54*
**Study B**	**MNB**	21.0% (41/195)	11.9% (248/2089)	11.2% (52/463)
	**Non-MNB**	7.4% (307/4127)	4.8% (327/6750)	1.4% (231/16965)
	**MNB %**	4.5	23.6	2.7
	*chi-sq*.	*46*.*43*	*129*.*53*	*274*.*82*
	*p-value*	*<0*.*001*	*<0*.*001*	*<0*.*001*
	*Odds Ratio*	*3*.*31*	*2*.*65*	*9*.*12*
	*95% CI*	*2*.*30–4*.*77*	*2*.*23–3*.*15*	*6*.*68–12*.*58*

DUCs: direct unequal cleavage. ICSI: Intracytoplasmic sperm injection**.** MNB**:** multinucleated blastomere.

### Developmental potential of DUC embryos

When using static embryo assessment, without time-lapse, a drop in morphologically “good” day 3 embryos was observed for DUC-1 embryos (27.4% vs. 56.4% for non-DUC, *p*<0.0001). However, the proportion of “good” embryos for DUC-2 (57.1%, *p* = .14) and DUC-3 (91.8%, *p* < .0001) were equal to or higher when compared to non-DUC embryos. This implies that DUC embryos would more likely be chosen for ET on D3 when evaluated statically without time-lapse. On the other hand, blastocyst formation rates of DUC embryos decreased proportionately from 40.2% for DUC-3, 18.8% for DUC-2, 8.2% for DUC-1 to 5.6% for DUC-Plus, compared to 61.0% in non-DUC embryos (all *p*<0.001) (S2 Fig). To predicts the probability of a good blastocyst (cryopreserved or transferred) given the DUC category, the MNB stage and the oocyte age, logistical regression was conducted in 8933 embryos from 926 blastocyst culture cycles. The odds ratios for these predictors were listed incrementally in S2 Table. The propensity of forming good blastocyst under best condition is 76.4% (21 Years, Non-DUC and Non-MNB), 0.1% in worst condition (46 years, DUC-Plus and MNB-1). (S3 Fig)

Time-lapse also provided details of blastomere behavior during blastocyst development. Most DUC daughter blastomeres did not participate in the embryo proper throughout compaction and cavitation. Blastomeres were excluded from the developing embryo and were clearly visible during embryo cavitation and subsequent BL formation (S1 and S2 Videos). Occasionally these blastomeres degenerated. The phenomena were not limited to DUC embryos, but were also observed in embryos with other abnormal division behavior (e.g. cell fusion, non-division or delayed division, and karyokinesis without cytokinesis).

### Clinical outcome of DUC embryos

**Fig 2:**
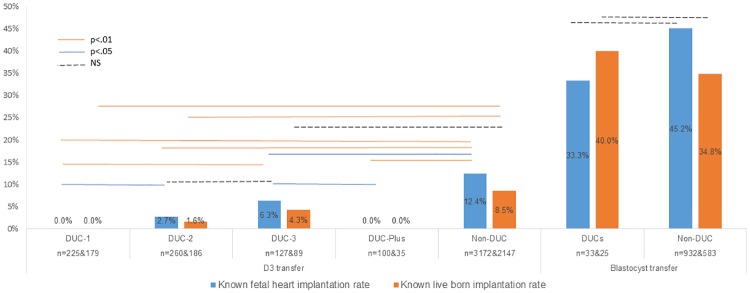
D3 and blastocyst transfer known implantation rate. Left: D3 transfer result. Right: Blastocyst transfer result (including frozen transfer cycles). DUC-1: direct unequal cleavage at 1^st^ cleavage; DUC-2: direct unequal cleavage at 2nd cleavage; DUC-3: direct unequal cleavage at 3^rd^ cleavage; DUC-Plus: DUC occurred more than once. Non-DUC: embryos without DUC. DUCs: all DUC embryos.

In D3 transfer, DUC embryos showed a significant decrease in known implantation rate (KID-FH): 6.3% (n = 127) in DUC-3; 2.7% (n = 260) in DUC-2; to zero in both DUC-1 (n = 225) and DUC-Plus (n = 100) compared to 12.4% in non-DUC embryos (n = 3172) (Fig 2. Left). The same trend was observed for known live birth rate (KID-LB): 4.3% (n = 89) in DUC-3; 1.6% (n = 186) in DUC-2; to zero in both DUC-1 (n = 179) and DUC-Plus (n = 35) compared to 8.5% in non-DUC embryos (n = 2147). None of the babies from DUC-2 (n = 5) or DUC-3 (n = 5) embryos showed any major birth defects.

For blastocyst transfer, all DUCs blastocysts (fresh and frozen) were combined for outcome analysis due to the small sample size. KID-FH rate was lower in DUC (33.3%, n = 34) vs. Non-DUC embryos (45.2%, n = 932) without reaching significance (p = 0.17, Fig 2. Right). KID- LB rates were similar between the DUCs (40%, n = 18) and Non-DUC (34.8%, n = 583, p = 0.59, Fig 2. Right). No known live births resulted from DUC-1 embryos transferred on day 3 or day 5.

### Chromosome analysis of DUC embryos

A total of 303 PGS/PGD cycles with 1434 embryos were included in this study. The ploidy results were classified as: euploid, aneuploid, complex abnormality (CxA, more than 1 chromosomal abnormality), chromosomal structure abnormality only, mosaicism, haploid (1n), or polyploidy (> = 3n) (S3 Table). The distribution of euploid, aneuploid and CxA among DUCs groups were summarized in Table 4. For non-DUC embryos, euploid rates were similar in D3 (45.6%, 159/349) and D5 biopsy embryos (51.4%, 171/333, *p* =.13), but dropped to 33.8% (169/500) in D6 biopsy embryos (n = 524, *p*<0.001). The euploid rate in D3 biopsied embryos increased gradually from DUC-1, DUC-2, DUC-3 to Non-DUC (13.3%, 19.5%, 33.3%, 45.6%, n = 446, p<0.001), conversely the CxA rate decreased from 83.3%, 51.2%, 38.9%, to 28.4% (p<0.001). All DUC embryos in D5/6 biopsy were combined for analysis due to sample size. DUCs blastocysts showed a similar euploid rate when compared to Non-DUC blastocysts on D5 (56.3%, n = 16 vs. 51.4%, n = 333, *p* = 0.57) and D6 (35.6%, n = 73 vs. 33.8%, n = 500, *p* = 0.79) biopsy (Table 5).

**Table 4:** Summary of preimplantation genetic screen results in day 3/5/6 biopsied DUC embryos.

Biopsy Day	DUCs	n	Euploid%	Aneuploid%	CxA%
**D3**	**DUC-1**	30	13.3%	3.3%	83.3%
	**DUC-2**	41	19.5%	29.3%	51.2%
	**DUC-3**	18	33.3%	27.8%	38.9%
	**DUC-Plus**	8	0.0%	25.0%	75.0%
	**Non-DUC**	349	45.6%	26.1%	28.4%
	*p*		<.001	10.0%	<.001
**D5**	**DUC-1**	2	50.0%	50.0%	0.0%
	**DUC-2**	5	60.0%	0.0%	40.0%
	**DUC-3**	7	42.9%	14.3%	42.9%
	**DUC-Plus**	2	100.0%	0.0%	0.0%
	**Non-DUC**	333	51.4%	26.4%	22.2%
**D6**	**DUC-1**	8	37.5%	50.0%	12.5%
	**DUC-2**	27	25.9%	40.7%	33.3%
	**DUC-3**	35	40.0%	22.9%	37.1%
	**DUC-Plus**	3	66.7%	0.0%	33.3%
	**Non-DUC**	500	33.8%	25.4%	40.8%

DUC-1: direct unequal cleavage at 1st cleavage; DUC-1Plus: DUC occur more than once in DUC-1 embryos; DUC-2: direct unequal cleavage at 2nd cleavage; DUC-2Plus: DUC occur more than once in DUC-2 embryos; DUC-3: direct unequal cleavage at 3rd cleavage; DUC-3Plus: DUC occur more than once in DUC-3 embryos; Non-DUC: embryos without DUC; DUC-Plus all: DUC occurred more than once (DUC-1Plus, DUC-2Plus and DUC-3Plus combined). Aneuploid: only 1 chromosome copy number error. CxA: complex abnormality, more than one chromosome error.

**Table 5:** Summary of preimplantation genetic screen results in day 3/5/6 biopsied DUC embryos (DUCs combined).

		n	Euploid%	Aneuploid%	CxA%
**D3 biopsy**	**DUCs**	97	18.6%	20.6%	60.8%
	**Non-DUC**	349	45.6%	26.1%	28.4%
	*chi-sq*.		*23*.*12*	*1*.*21*	*34*.*96*
	*p-value*		*0*.*00*	*0*.*18*	*0*.*00*
**D5 biopsy**	**DUCs**	16	56.3%	12.5%	31.3%
	**Non-DUC**	333	51.4%	26.4%	22.2%
	*chi-sq*.		*0*.*15*	*1*.*55*	*0*.*71*
	*p-value*		*0*.*93*	*0*.*46*	*0*.*70*
**D6 Biopsy**	**DUCs**	73	35.6%	31.5%	32.9%
	**Non-DUC**	500	33.8%	25.4%	40.8%
	*chi-sq*.		*0*.*09*	*1*.*23*	*1*.*67*
	*p-value*		*0*.*95*	*0*.*54*	*0*.*43*

DUCs: All direct equal cleavage. Non-DUC: embryos without DUC. Aneuploid: only 1 chromosome copy number error. CxA: complex abnormality, more than one chromosome error.

## Discussion

### Incidence

Our analysis is the first known large study which classifies DUCs based on the cleavage stages. The incidence of DUC-1 in our study was 9.8% which was within the previously reported range (8.3%-26%) [10, 14–16]. In contrast, DUC-2 incidence of 9.1% was much lower than reported elsewhere (17–18%) [5, 14]. The high DUC-1 incidence reported could be due to the incorrect identification of large fragment as blastomere. The higher incidence of DUC-1 was 14.3% in human [21] and 14.1% in bovine [22] 3PN embryos. Time-lapse allows for the correct identification of PN to be achieved. In our study, all DUC embryos were detected base on clearly visible nucleus of daughter blastomeres, progressive tracking of blastomere divisions, and confirmed by one senior embryologist.

Observation of DUCs after the 3rd cleavage stage is difficult due to the high cell number, small cell size and the onset of compaction. The reduction of DUCs after second cleavage might indicate different DUC mechanisms in later cleavage stage embryos. It also indicates the absence of the fully functional cell cycle checkpoints in early cleavage stage embryos before embryonic genome activation (EGA)[17].

The high prevalence of DUCs in human IVF cycles cannot be ignored in routine IVF procedures. These abnormally dividing embryos are prone to be selected as “good” embryos by classic static embryo assessment. Correct detection of DUCs requires time-lapse imaging, thus ratifying the critical necessity of time-lapse incubation systems in clinical embryology.

### DUCs correlation with gamete age, source and sperm maturity

Previously published studies on human DUCs have been based on abnormally fertilized oocytes or small sample sizes where gamete source or other clinical factors were not reported [6, 14, 16]. Our results show no significant differences in the incidence of DUCs between the maternal or paternal age groups or oocyte source (Table 1). The use of cryopreserved spermatozoa had no impact on the incidence of DUCs. On the contrary, the incidence of DUC-1 (the most severe abnormality) increased in embryos using epididymal and testicular sperm compared to ejaculated sperm. Since the same culture system was used, the possible role of culture condition (medium) could not be investigated here. However, our preliminary data of using different commercial media reveal similar DUC incidence compared to C1/C2 (results not shown).

The paternal inheritance of the centrosome and its centriole components has been firmly established in humans [7] and most animals with the exception of mice and other rodents, where they are maternally inherited [23, 24]. Both proximal and distal centrioles are present in spermatids, where distal centrioles progressively degenerate during spermiogenesis [25]. During gametogenesis, centrioles remain in spermatozoa but have lost most of the pericentriolar material. During fertilization the male gamete contributes two centrioles that organize a functional zygotic centrosome after recruiting centrosomal proteins from the oocyte’s cytoplasm[26]. After sperm incorporation into the oocyte, a sperm aster is formed from the proximal centriole. The sperm centriole duplicates during the pronuclear stage and separates after syngamy to serve as the mitotic center from the first cleavage division up to the blastocyst stage [27, 28]. During that period the maternal centrosome is not functional [29].

Improper centrosomal inheritance or dysfunction of the sperm centriole may be associated with cleavage irregularities and/or abnormal embryonic development [24, 27, 30, 31]. Most polyspermic embryos result in multipolar spindle formation and multipolar mitosis. This kind of abnormal spindle formation is unlikely the cause of DUC in humans as we observed a very low incidence (1/196, S3 Table) of triploid in biopsied DUC embryos. Additionally, a very low incidence of DUCs were observed in parthenogenetic bovine embryos [22].

The higher DUC-1 incidence in embryos using epididymal sperm may point to the incomplete degeneration of distal sperm centrioles. No studies have investigated the possible effects of the sperm’s centriole–centrosome complex on consequent embryo development [32]. However, studies on sperm centrosome pathology in human populations have been published [24, 33]. The incidence of DUC-1 might be used as an indirect indicator of sperm quality (sperm centriole function), as no functional centrosomal tests are currently available. Since maternal centrosomal proteins play an important role during spindle formation, the DUC occurrence may also be correlated with ooplasm maturity. Further studies are necessary to clarify these hypothetical correlations.

One of the possible hypothesis underlying DUCs is formation of multipolar spindles through the introduction of either incomplete, defective or supernumerary centrioles by defective sperm.

### DUCs and fertilization methods

Our data indicates a correlation between conventional insemination and DUC-1 incidence. One possible explanation is the occurrence of occult polyspermic fertilization [22]. The occurrence of undetected 3PN embryos fertilized by diploid sperm (dispermic) or failure of 2^nd^ PB extrusion resulting in diploid oocytes (digynic) might increase the incidence of DUCs [21]. No differences have been reported in male centrosome behavior in oocytes fertilized by ICSI or insemination [24]. Additionally, there is no supporting evidence that centrosome dysfunction increases after ICSI [24], even in men with severe sperm parameters.

### Multinucleated embryos are prone to DUCs occurrence

Multinucleation is a common phenomenon in human embryo development *in vitro* and plays an important role in embryo assessment. It is correlated with increased fragmentation, lower blastocyst formation rate [34], higher chromosomal abnormalities [35], and impaired implantation [36]. In our study, MNB occurred in 39% of embryos. In previously published studies, MNB occurred in over 70% of cycles and in 30% of embryos [36, 37]. The incidence of MNB increased with suboptimal oocyte maturity in shorter stimulation protocols with higher FSH doses [37] and higher oocyte yields [36, 37]. However, no correlation between MNB rate and female age have been reported [36]. The higher MNB frequency in our study may be the result of close monitoring of embryos using time-lapse microscopy, compared to the previously reported study[38].

In our study, a strong correlation between DUCs and multinucleation was revealed. The risk of DUC occurrence in multinucleated embryos was 2.5–3.1 times higher than in mononucleated embryos. This is the first report revealing a strong correlation between embryo multinucleation and DUCs occurrence. The mechanism of multinucleation formation was not clear. During the 1^st^ cleavage, multinucleation most likely occurred due to chromosome segregation errors and/or cytokinesis failure (endomitosis) [39, 40]. The occurrence of MNB in embryos proceeding through the 2^nd^ and 3^rd^ cleavages may be due to the abnormal karyokinesis, as well as chromosome segregation errors and/or other mitotic errors [41]. Multinucleation was also associated with increased aneuploidy and chromosomal abnormalities [42] along with abnormalities in DNA synthesis [43]. One possible explaination for the high incidence of DUCs in MNB embyos or blastomeres could be the DNA damage in multinuclei-bearing cells, causes centriole over-replication or endomitosis forming multipolar spindle which results in multipolar mitosis. Destouni et al. proposed three models underlying heterogoneic division (DUC-1): concurrent operation or residual meiotic spindles, loss of the gonomeric spindle pole integrity and endomitotic cycles [40]. In addition, multinucleation rates following DUC-2 and DUC-3 were not significatly different from that of Non-DUC embryos.

In early cleavage stages, spindle assembly checkpoints (SAC) are not truly functional and become fully functional after embryonic genome activation (EGA) [17, 44]. Fully functional SAC might reduce the incidence of MNB and DUCs in later cell stages. Further studies are necessary to elucidate mechanisms of MNB formation and subsequent abnormal divisions.

### Developmental potential and clinical outcome

Previous studies have shown a clear correlation between the occurrence of DUCs, impaired embryo development, and implantation potential in both animals [22] and humans [5, 9, 10, 12–14, 17]. Our study confirms that DUCs in early stages strongly correlate with impaired blastocyst formation, implantation, and clinical outcome. Meanwhile later stage DUCs have a milder impact. Based on S2 Table, DUC-1, DUC-Plus and MNB at 1 cell stage were most detrimental to blastocyst development.

The correlation between impaired developmental potential and early stage DUCs may reflect the higher portions of cells have been affected and are being subjected to apoptosis which was is caused by the not-fully functional SAC during EGA [17]. Also, a recent study uncovered bovine 2PN can segregate entire parental genome into different cell lineage though heterogoneic division (DUC-1) causing chimerism and mixoploidy[40]. These mechanisms may also explain why DUC-1 KID-FH rates in D3 transfers dropped to 0%. Published reports indicate an implantation rate of 3.7% in DUC-1 and DUC-2 embryos [14]. One possible explanation was the small sample size (n = 639) and their inability to correctly detect DUCs based only on cell/fragment size and the dark field image resolution.

### Chromosome analysis of DUC embryos

High incidence of aneuploidy was found in human polyspermic DUC embryos [11, 45]. No chromosomal studies of diploid human DUC embryo have been reported to date. This is the first study to describe the chromosomal constitution of 2PN DUC embryos. In D3 biopsied DUC embryos, euploidy rates increased (from 1^st^ to 3^rd^ cleavage), while complex abnormality rates decreased according to the DUC stage (Table 4). A recent report indicated an association between the increase in blastomere number on D3 with higher aneuploidy rates [46]. This observation may in fact be due to DUCs. The earlier the onset stage of DUCs, the lower the chance of euploidy and the higher the chance of complex chromosomal abnormalities. Destouni et al. confirmed heterogoneic division (DUC-1) can cause cleavage-stage chimerism and mixoploidy in normal fertilized bovine embryos [40]. The high incidence of aneuploidy in DUC embryos suggests they should be deselected for transfer (especially DUC-1).

Chatzimeletiou et al. proposed that a major pathway leading to postzygotic chromosomal abnormalities was the formation of binucleated blastomeres with two centrosomes which resulted in either a bipolar spindle or division into two tetraploid blastomeres [31]. Other alternatives were the development of multipolar spindles, chromosome malsegregation and chromosomal chaos [17].

Shown in this study, high aneuploidy rates in DUC embryos and close association between multinucleation and DUCs provide strong evidence of possible links between MNB and genetic abnormality.

Our data shown good DUC blastocysts have a comparable euploid rate as Non-DUC. This suggests that extending embryo culture to blastocyst stage allows the “self-correction” event to occur, which eliminates the abnormally divided cells in DUC embryos, DUC embryos will be deselected by not reaching blastocyst stage.

These finding have some limitations. As for one, genetic diagnoses were based on varying platforms (FISH, aCGH and SNP array). Second, mosaicism and polyploidy may not reflect the real incidence due to the limited ability of aCGH to detect polyploidy and mosaicism. Moreover, only good embryos were biopsied and partially analyzed. Another concern is the possible incidence of mosaicism caused by DUCs, which could have overestimated the euploid rate in this study. However, large studies have estimated a 5–7% error caused by mosaicism [47, 48] when using multiple probe FISH.

### Embryo “self- correction”

In our observation, DUC daughter blastomeres extrusion from the embryo proper during compaction seemed to be a common and necessary occurrence for reaching good blastocysts[49]. Delayed or abnormal divisional behavior (cell fusion, karyokinesis without cytokinesis) and degeneration were observed in daughter DUC blastomeres. In general, DUC daughter cells will arrest and/or degenerate and will be extruded from the blastocyst (S2 and S3 Videos) which suggest the theory of embryo “self-correction”. Although similar of euploid rate in good blastocyst between DUCs and non-DUC supports this hypothesis, chromosomal content of extruded blastomeres and corresponding blastocyst need to be further investigated.

Cell relocation and exclusion from the embryo proper during blastulation might be related to E-cadherin re-localization [50]. E-cadherin distribution, located in the cytoplasm of early human embryo, is stage-dependent. This protein is distributed on the membranes in the areas of cell to cell contact after embryo genome activation and is important for embryo compaction [50]. The occurrence of DUCs may disturb the relocation of E-Cadherin to the cell membranes, within DUC blastomeres, resulting in cell extrusion. A lower implantation rate of the “rescued” DUC blastocysts can be linked to the reduction of the available cellular mass that forms blastocysts as well as an overall lower blastomere number. The additional evidence of “self- correction” comes from the study of polyspermic embryos where DUC-1 was believed to be involved in the occasional correction of abnormal ploidy [11, 51, 52]. In a study by Kola, I., et al. on 29 human 3PN embryos: 4 were diploid after cleaving into 2-cells plus an extrusion, 7 were triploid and the remaining were complex abnormal [11]. Our preliminary chromosomal analysis of extruded blastomeres and matching trophectoderm cells revealed a higher incidence of aneuploidy in extruded cells versus corresponding trophectoderm cells [53].

DUC blastocysts have similar euploidy rates but their viability seems to be impaired based upon our implantation data. Although live births have resulted from the transfer of DUC-3 and DUC-2 embryos, no known live births have resulted from the transfer of DUC-1 embryos (n = 232). All babies born from DUC embryos were free of major and minor birth defects.

### Clinical application

**Fig 3:**
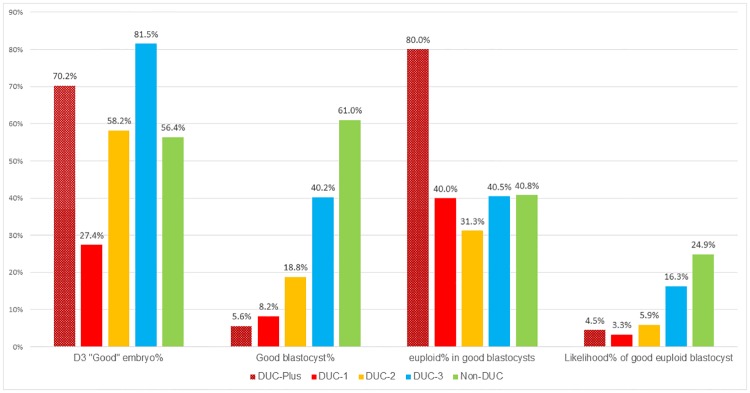
Estimated likelihood of DUC embryos development into good euploid blastocyst. DUC-1: direct unequal cleavage at 1^st^ cleavage; DUC-2: direct unequal cleavage at 2nd cleavage; DUC-3: direct unequal cleavage at 3^rd^ cleavage; DUC-Plus: DUC occurred more than once. Non-DUC: embryos without DUC.

The prevalence and detrimental effects of DUCs on embryo development, chromosomal constitution and clinical outcome highlights the importance of uninterrupted time-lapse monitoring of IVF embryos. Due to the high overall DUC prevalence (26.1%), the chance of transferring a DUC embryo was approximately 1 in 4 without TLM for D3 transfer. The D3 implantation rate could be improved simply through the de-selection of DUC embryos. Based on our results, DUC embryos should not be transferred, especially DUC-1 and DUC-2. These embryos should be cultured to the blastocyst stage for transfer or cryopreservation. Fig 3. lists the likelihood of DUC embryos to develop into a good quality euploid blastocyst based on our data: 4.5% in DUC-Plus, 3.3% in DUC-1, 5.9% in DUC-2, 16.3% in DUC-3 and 24.9% in Non-DUC embryos. Clearly, the viability of DUC embryos is compromised which lead to low implantation rates. Thus, identification and deselection of early cleavage stage DUC embryos should improve the success of single embryo transfers. DUC blastocysts have similar euploidy rates but their viability seems to be impaired based upon our implantation data.

## Conclusion

Blastocyst formation, implantation potential and euploid rate were significantly reduced in embryos exhibiting direct unequal cleavage when compared to control cohort. The observed reductions were inversely proportional to advanced cleavage stages. Embryos exhibiting DUCs in the first two cleavage stages should be deselected from D3 transfers, but may still be considered for blastocyst transfer if they reach the blastocyst stage and exhibit good morphology.

## Supporting Information

S1 Minimal Data Set (XLSX)

S1 Fig DUCs distribution in ICSI and IVF cycles. IVF: In vitro fertilization; ICSI: Intracytoplasmic sperm injection; <4 2PN embryos: cycles with less than four two-pronuclear embryos; ≥50% DUC embryos: In cycle with 4 or more 2PN, more than half embryos exhibiting direct unequal cleavage; <50% DUC embryos: In cycles with 4 or more 2PN, less than half embryos exhibiting direct unequal cleavage; DUC-free: In cycles with 4 or more 2PN, none exhibiting direct unequal cleavage.
(TIF)

S2 Fig D3 static morphological assessment and developmental potential of DUC embryos. Left: Proportion of “Good” embryos (8 or more cells and less than 20% fragmentation on day 3) when embryos accessed on day 3 by static morphologic criteria. Right: Good blastocyst (2BB higher) formation rate in embryos from blastocysts transfer cycles only. DUC-1: direct unequal cleavage at 1^st^ cleavage; DUC-2: direct unequal cleavage at 2nd cleavage; DUC-3: direct unequal cleavage at 3^rd^ cleavage; DUC-Plus: DUC occurred more than once. Non-DUC: embryos without DUC.
(TIF)

S3 Fig Prediction Profiler showing best (top) and worst (bottom) conditions for Good Blastocyst formation. DUC-1: direct unequal cleavage at 1^st^ cleavage; DUC-2: direct unequal cleavage at 2nd cleavage; DUC-3: direct unequal cleavage at 3^rd^ cleavage; DUC-Plus: DUC occurred more than once. Non-DUC: embryos without DUC. MNB-1: multinucleated blastomere presented in 1-cell stage; MNB-2: multinucleated blastomere presented in 2-cell stage; MNB-4: multinucleated blastomere presented in 4-cell stage; MNB-8: multinucleated blastomere presented in 8-cell stage; Non-MNB: None multinucleated blastomere presented in early stage. Good blastocyst (2BB higher) formation rate in embryos from blastocysts culture cycles only.
(TIF)

S1 Table Incidence of multinucleated blastomere(MNB) following DUCs at different stages. DUC: Direct unequal cleavage, DUCs: all DUC embryos, Non-DUC: embryos without DUC.
(XLSX)

S2 Table Odds Ratios for DUCs, MNBs and Oocyte Age in Good Blastocyst Formation. DUC-1: direct unequal cleavage at 1st cleavage; DUC-2: direct unequal cleavage at 2nd cleavage; DUC-3: direct unequal cleavage at 3rd cleavage; DUC-Plus: DUC occurred more than once. Non-DUC: embryos without DUC; MNB-1: multinucleated blastomere at 1-cell stage; MNB-2: multinucleated blastomere presented at 2-cell stage; MNB-4: multinucleated blastomere presented at 4-cell stage; MNB-8: multinucleated blastomere presented at 8-cell stage; Non-MNB: None multinucleated blastomere presented in early cleavage stage. *: statistically significant.
(XLSX)

S3 Table Preimplantation genetic screen results in DUC embryos (Day 3/5/6 biopsy). DUC-1: direct unequal cleavage at 1st cleavage; DUC-1Plus: DUC occur more than once in DUC-1 embryos; DUC-2: direct unequal cleavage at 2nd cleavage; DUC-2Plus: DUC occur more than once in DUC-2 embryos; DUC-3: direct unequal cleavage at 3rd cleavage; DUC-3Plus: DUC occur more than once in DUC-3 embryos; Non-DUC: embryos without DUC; DUC-Plus all: DUC occurred more than once (DUC-1Plus, DUC-2Plus and DUC-3Plus combined). Aneuploid: only 1 chromosome copy number error. CxA: complex abnormality, more than one chromosome error.
(XLSX)

S1 Video DUC-1. Direct uneven cleavage at first cleavage(DUC-1) resulted in 3 blastomeres with clear visible nuclei. Each daughter blastomeres divided twice resulting in 9 cells.
(AVI)

S2 Video DUC-2 Extrusion. Direct uneven cleavage at second cleavage (DUC-2). One blastomere in 2-cell stage divided into 3 daughter cells forming 5-cell embryo. After compaction, they were extruded from blastocyst formation (4 o’clock position).
(AVI)

S3 Video DUC-3 partially extrusion. Direct uneven cleavage at the third cleavage (DUC-3), resulted in 9-cell embryo. DUC-3 daughter blastomeres were partially extruded during compaction (DUCS blastomeres at 6 o’clock position).
(AVI)
