# Supplementary figures and images for: Direct Unequal Cleavages: Embryo Developmental Competence, Genetic Constitution and Clinical Outcome
Source: PLoS One. 2016 Dec 1;11(12):e0166398. doi: 10.1371/journal.pone.0166398 (PMC5132229; doi:10.1371/journal.pone.0166398)

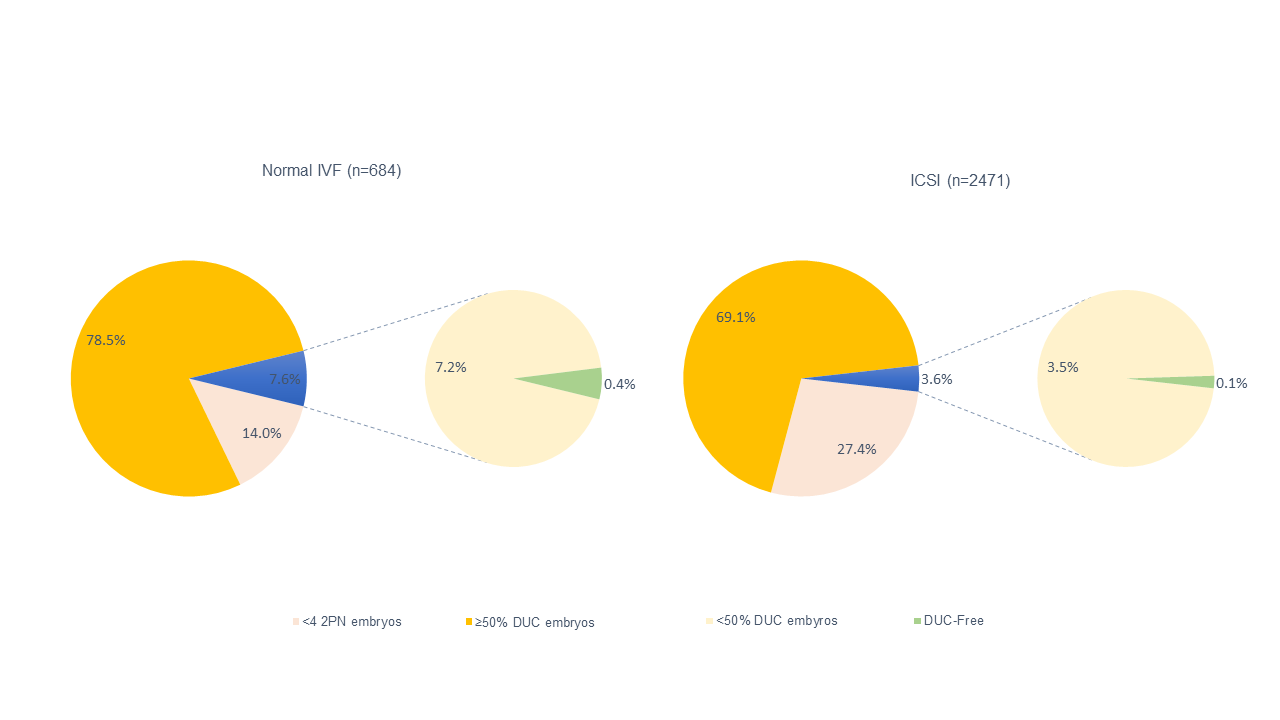

Supplement: S1 Fig — IVF: In vitro fertilization; ICSI: Intracytoplasmic sperm injection; <4 2PN embryos: cycles with less than four two-pronuclear embryos; ≥50% DUC embryos: In cycle with 4 or more 2PN, more than half embryos exhibiting direct unequal cleavage; <50% DUC embryos: In cycles with 4 or more 2PN, less than half embryos exhibiting direct unequal cleavage; DUC-free: In cycles with 4 or more 2PN, none exhibiting direct unequal cleavage. (TIF) [file pone.0166398.s002.tif]

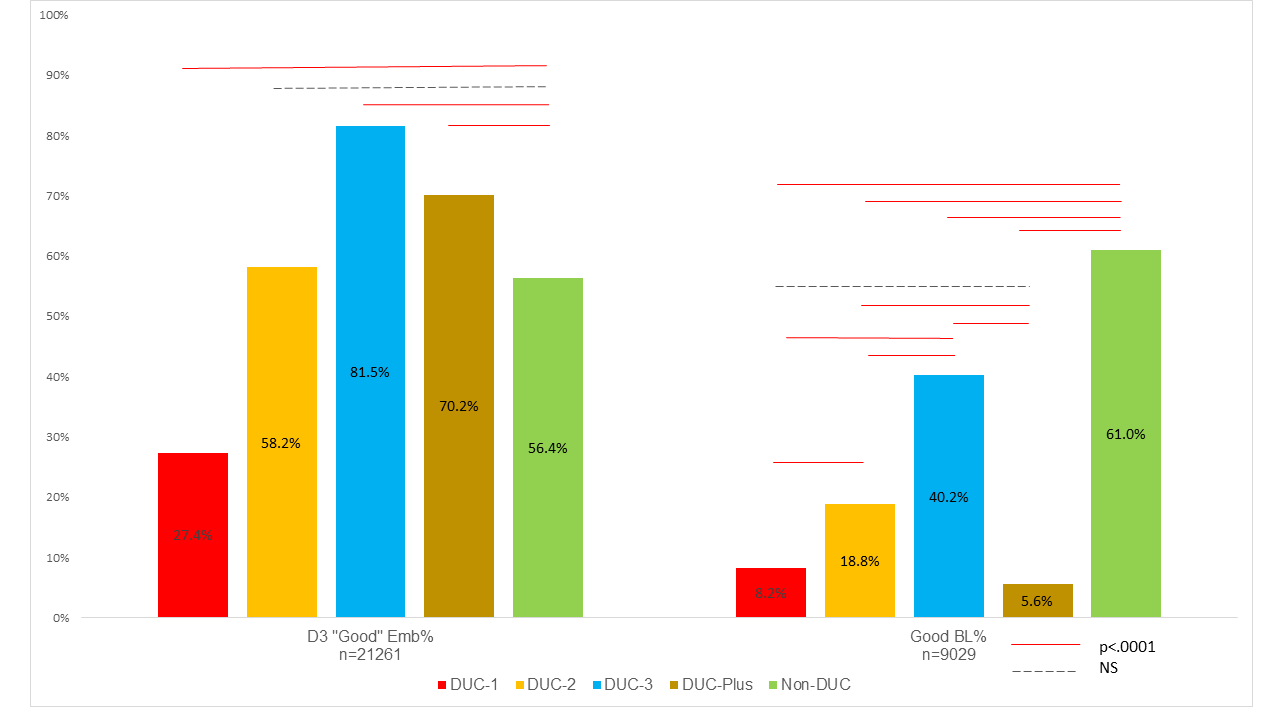

Supplement: S2 Fig — Left: Proportion of “Good” embryos (8 or more cells and less than 20% fragmentation on day 3) when embryos accessed on day 3 by static morphologic criteria. Right: Good blastocyst (2BB higher) formation rate in embryos from blastocysts transfer cycles only. DUC-1: direct unequal cleavage at 1st cleavage; DUC-2: direct unequal cleavage at 2nd cleavage; DUC-3: direct unequal cleavage at 3rd cleavage; DUC-Plus: DUC occurred more than once. Non-DUC: embryos without DUC. (TIF) [file pone.0166398.s003.tif]

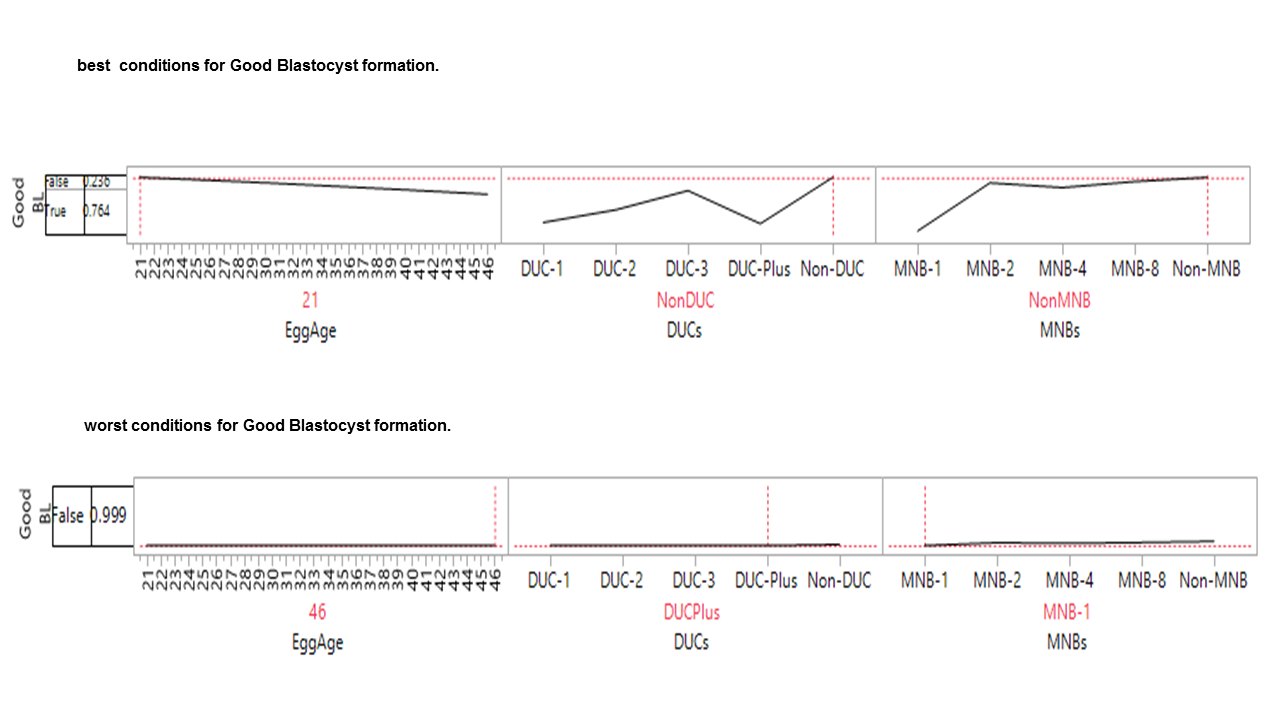

Supplement: S3 Fig — DUC-1: direct unequal cleavage at 1st cleavage; DUC-2: direct unequal cleavage at 2nd cleavage; DUC-3: direct unequal cleavage at 3rd cleavage; DUC-Plus: DUC occurred more than once. Non-DUC: embryos without DUC. MNB-1: multinucleated blastomere presented in 1-cell stage; MNB-2: multinucleated blastomere presented in 2-cell stage; MNB-4: multinucleated blastomere presented in 4-cell stage; MNB-8: multinucleated blastomere presented in 8-cell stage; Non-MNB: None multinucleated blastomere presented in early stage. Good blastocyst (2BB higher) formation rate in embryos from blastocysts culture cycles only. (TIF) [file pone.0166398.s004.tif]
